# The Heart in Early Life

**Published:** 1915-06

**Authors:** 


**The Heart in Early Life.** By G. A. Sutherland, M.D. Pp. xvi., 211. London: Oxford Medical Publications. 1914. Price, 6s. net.

Dr. Sutherland has brought three essential qualifications for authorship into co-operation in the writing of this book: wide experience, a critical judgment, and a faculty for setting down his thoughts in a readable form. His basic argument is this, that the disorders and diseases of the heart as seen in early life are distinct in important respects from those of maturer years. In particular it should be noted that the action of the heart in early life is more susceptible of modification by extraneous influences, that it is a growing organ, and that during childhood rheumatic infection is actively and progressively injuring the heart and laying the foundations of the chronic valvular diseases of adult life. To the study of these particular features Dr. Sutherland has applied not only eyes and ears but also the graphic methods of the newer cardiology, but without giving them more prominence than they deserve. No one need fear that the book is too technical to be interesting. It is easily read, and the principles that Dr. Sutherland seeks to emphasise are driven home by suitable examples.

